# Supplementary material for: Allosteric regulation and crystallographic fragment screening of SARS-CoV-2 NSP15 endoribonuclease
Source: Nucleic Acids Res. 2023 Apr 28;51(10):5255–70. doi: 10.1093/nar/gkad314 (PMC10250223; doi:10.1093/nar/gkad314)
Supplement: gkad314_Supplemental_Files [file gkad314_supplemental_files.zip › Supplementary datasets.zip!/183/Refine_4/01-BUSTER/Cycle-2/shell.01/R_Rfree.html]

# Rxpct factors in resolution bins at cycle 0


|  |  |  |  |  |  |  |  |  |
| --- | --- | --- | --- | --- | --- | --- | --- | --- |
|  | **<d> (Å)**  Rxpct\_Work | Rxpct\_Free | Rxpct\_All | |  |  |  |  |
|  | 14.0455 | 0.3079 | 0.3210 | 0.3085 |  |
|  | 8.1092 | 0.2169 | 0.2392 | 0.2182 |  |
|  | 6.2814 | 0.2448 | 0.2340 | 0.2442 |  |
|  | 5.3087 | 0.1967 | 0.1965 | 0.1967 |  |
|  | 4.6818 | 0.1638 | 0.1752 | 0.1644 |  |
|  | 4.2349 | 0.1663 | 0.1583 | 0.1659 |  |
|  | 3.8955 | 0.1766 | 0.1886 | 0.1772 |  |
|  | 3.6265 | 0.1867 | 0.2180 | 0.1883 |  |
|  | 3.4065 | 0.1932 | 0.2003 | 0.1936 |  |
|  | 3.2223 | 0.1847 | 0.1982 | 0.1855 |  |
|  | 3.0650 | 0.1898 | 0.1947 | 0.1900 |  |
|  | 2.9287 | 0.1882 | 0.2020 | 0.1888 |  |
|  | 2.8091 | 0.1921 | 0.2183 | 0.1933 |  |
|  | 2.7031 | 0.1988 | 0.2000 | 0.1989 |  |
|  | 2.6082 | 0.2162 | 0.2294 | 0.2170 |  |
|  | 2.5227 | 0.2186 | 0.2304 | 0.2192 |  |
|  | 2.4450 | 0.2288 | 0.2439 | 0.2295 |  |
|  | 2.3741 | 0.2463 | 0.2476 | 0.2464 |  |
|  | 2.3091 | 0.2713 | 0.2917 | 0.2722 |  |
|  | 2.2491 | 0.3090 | 0.3053 | 0.3089 |  |
|  | **Overall** | 0.2018 | 0.2099 | 0.2022 |  |

# Rxpct factors in resolution bins at cycle 1


|  |  |  |  |  |  |  |  |  |
| --- | --- | --- | --- | --- | --- | --- | --- | --- |
|  | **<d> (Å)**  Rxpct\_Work | Rxpct\_Free | Rxpct\_All | |  |  |  |  |
|  | 14.0455 | 0.3079 | 0.3206 | 0.3084 |  |
|  | 8.1092 | 0.2171 | 0.2394 | 0.2184 |  |
|  | 6.2814 | 0.2447 | 0.2339 | 0.2442 |  |
|  | 5.3087 | 0.1975 | 0.1944 | 0.1974 |  |
|  | 4.6818 | 0.1653 | 0.1759 | 0.1659 |  |
|  | 4.2349 | 0.1681 | 0.1592 | 0.1676 |  |
|  | 3.8955 | 0.1785 | 0.1894 | 0.1790 |  |
|  | 3.6265 | 0.1890 | 0.2218 | 0.1907 |  |
|  | 3.4065 | 0.1951 | 0.2007 | 0.1954 |  |
|  | 3.2223 | 0.1869 | 0.2021 | 0.1877 |  |
|  | 3.0650 | 0.1922 | 0.1973 | 0.1924 |  |
|  | 2.9287 | 0.1897 | 0.2039 | 0.1904 |  |
|  | 2.8091 | 0.1936 | 0.2199 | 0.1948 |  |
|  | 2.7031 | 0.1995 | 0.2001 | 0.1995 |  |
|  | 2.6082 | 0.2155 | 0.2322 | 0.2165 |  |
|  | 2.5227 | 0.2178 | 0.2292 | 0.2184 |  |
|  | 2.4450 | 0.2276 | 0.2407 | 0.2282 |  |
|  | 2.3741 | 0.2456 | 0.2484 | 0.2457 |  |
|  | 2.3091 | 0.2710 | 0.2863 | 0.2717 |  |
|  | 2.2491 | 0.3105 | 0.3100 | 0.3104 |  |
|  | **Overall** | 0.2029 | 0.2107 | 0.2033 |  |

# Rxpct factors in resolution bins at cycle 2


|  |  |  |  |  |  |  |  |  |
| --- | --- | --- | --- | --- | --- | --- | --- | --- |
|  | **<d> (Å)**  Rxpct\_Work | Rxpct\_Free | Rxpct\_All | |  |  |  |  |
|  | 14.0455 | 0.3080 | 0.3206 | 0.3085 |  |
|  | 8.1092 | 0.2173 | 0.2396 | 0.2186 |  |
|  | 6.2814 | 0.2450 | 0.2341 | 0.2445 |  |
|  | 5.3087 | 0.1984 | 0.1937 | 0.1981 |  |
|  | 4.6818 | 0.1666 | 0.1762 | 0.1671 |  |
|  | 4.2349 | 0.1694 | 0.1601 | 0.1689 |  |
|  | 3.8955 | 0.1796 | 0.1893 | 0.1800 |  |
|  | 3.6265 | 0.1903 | 0.2239 | 0.1920 |  |
|  | 3.4065 | 0.1959 | 0.2020 | 0.1962 |  |
|  | 3.2223 | 0.1879 | 0.2040 | 0.1888 |  |
|  | 3.0650 | 0.1930 | 0.1967 | 0.1931 |  |
|  | 2.9287 | 0.1902 | 0.2043 | 0.1908 |  |
|  | 2.8091 | 0.1941 | 0.2197 | 0.1952 |  |
|  | 2.7031 | 0.1995 | 0.2010 | 0.1996 |  |
|  | 2.6082 | 0.2149 | 0.2336 | 0.2160 |  |
|  | 2.5227 | 0.2174 | 0.2281 | 0.2180 |  |
|  | 2.4450 | 0.2269 | 0.2403 | 0.2275 |  |
|  | 2.3741 | 0.2453 | 0.2479 | 0.2454 |  |
|  | 2.3091 | 0.2707 | 0.2840 | 0.2713 |  |
|  | 2.2491 | 0.3112 | 0.3124 | 0.3112 |  |
|  | **Overall** | 0.2036 | 0.2112 | 0.2040 |  |

# Rxpct factors in resolution bins at cycle 3


|  |  |  |  |  |  |  |  |  |
| --- | --- | --- | --- | --- | --- | --- | --- | --- |
|  | **<d> (Å)**  Rxpct\_Work | Rxpct\_Free | Rxpct\_All | |  |  |  |  |
|  | 14.0455 | 0.3090 | 0.3313 | 0.3099 |  |
|  | 8.1092 | 0.2355 | 0.2689 | 0.2375 |  |
|  | 6.2814 | 0.2628 | 0.2960 | 0.2645 |  |
|  | 5.3087 | 0.2358 | 0.2802 | 0.2378 |  |
|  | 4.6818 | 0.2060 | 0.2242 | 0.2070 |  |
|  | 4.2349 | 0.2096 | 0.1889 | 0.2085 |  |
|  | 3.8955 | 0.2085 | 0.2278 | 0.2094 |  |
|  | 3.6265 | 0.2175 | 0.2716 | 0.2203 |  |
|  | 3.4065 | 0.2131 | 0.2342 | 0.2142 |  |
|  | 3.2223 | 0.2060 | 0.2195 | 0.2067 |  |
|  | 3.0650 | 0.2029 | 0.1917 | 0.2024 |  |
|  | 2.9287 | 0.1958 | 0.1912 | 0.1956 |  |
|  | 2.8091 | 0.1961 | 0.2136 | 0.1969 |  |
|  | 2.7031 | 0.2022 | 0.2164 | 0.2029 |  |
|  | 2.6082 | 0.2103 | 0.2240 | 0.2111 |  |
|  | 2.5227 | 0.2084 | 0.2210 | 0.2092 |  |
|  | 2.4450 | 0.2110 | 0.2299 | 0.2119 |  |
|  | 2.3741 | 0.2236 | 0.2291 | 0.2238 |  |
|  | 2.3091 | 0.2425 | 0.2497 | 0.2428 |  |
|  | 2.2491 | 0.3152 | 0.3145 | 0.3152 |  |
|  | **Overall** | 0.2209 | 0.2370 | 0.2217 |  |
